# Interaction between the exchanged Mn^2+^ and Yb^3+^ ions confined in zeolite-Y and their luminescence behaviours

**DOI:** 10.1038/srep46219

**Published:** 2017-04-10

**Authors:** Shi Ye, Jiayi Sun, Xiong Yi, Yonggang Wang, Qinyuan Zhang

**Affiliations:** 1 State Key Lab of Luminescent Materials and Devices, and Guangdong Provincial Key Laboratory of Fiber Laser Materials and Applied Techniques, South China University of Technology, Guangzhou 510641, China; 2 High Pressure Synergetic Consortium (HPSynC), Geophysical Laboratory, Carnegie Institution of Washington, Argonne, Illinois 60439, USA

## Abstract

Luminescent zeolites exchanged with two distinct and interacted emissive ions are vital but less-studied for the potential applications in white light emitting diodes, solar cells, optical codes, biomedicine and so on. Typical transition metal ion Mn^2+^ and lanthanide ion Yb^3+^ are adopted as a case study via their characteristic transitions and the interaction between them. The option is considered with that the former with d-d transition has a large gap between the first excited state ^4^T_1_ and the ground state ^6^A_1_ (normally >17,000 cm^−1^) while the latter with *f-f* transition has no metastable excited state above 10,000 cm^−1^, which requires the vicinity of these two ions for energy transfer. The results of various characterizations, including BET measurement, photoluminescence spectroscopy, solid-state NMR, and X-ray absorption spectroscopy, etc., show that Yb^3+^ would preferably enter into the zeolite-Y pores and introduction of Mn^2+^ would cause aggregation of each other. Herein, cation-cation repulsion may play a significant role for the high valence of Mn^2+^ and Yb^3+^ when exchanging the original cations with +1 valence. Energy transfer phenomena between Mn^2+^ and Yb^3+^ occur only at elevated contents in the confined pores of zeolite. The research would benefit the design of zeolite composite opto-functional materials.

Zeolite is a kind of significant material in the application of catalysis, absorption and ion-exchanging, owing to its unique periodic microporous structure. The most commonly used zeolite is aluminosilicate, the framework of which is composed of AlO_4_ and SiO_4_ tetrahedra. It can also be viewed as SiO_2_ with some SiO_4_ tetrahedron substituted by AlO_4_, resulting in electronegativity of the framework. It can be balanced by the cations attaching to the framework in the pores, such as Na^+^, K^+^, Mg^2+^, Ca^2+^, NH_4_^+^, etc. These cations are always easy to be exchanged with external ions. And the number and sites of the cations strongly affect the properties of the zeolites. Among all kinds of zeolites, zeolite-Y shows the faujasite (FAU) structure, whose pore diameter is about 7.4 Å as the aperture is defined by a 12-member oxygen ring resulting in a larger cavity diameter of 12 Å^1^.

The unique structure of zeolites is appealing for numerous applications besides catalysis and absorption. For instance, zeolite-Y are utilized as excellent hosts for luminescent materials because of their strong ability of separating the luminescent centers, thus, reducing the concentration quenching^2^,^3^. Therefore, it provides a superb chemical environment for luminescent centers due to its outstanding thermal and chemical stability, such as rare earth ions^4^,^5^,^6^, quantum dots^7^,^8^,^9^,^10^,^11^,^12^, and noble metal Ag^13^,^14^,^15^. These composite materials have potential applications in white light emitting diodes (WLEDs)^16^, solar cells^17^, optical code^18^, biomedicine^19^,^20^ and so on. Luminescent centers may be introduced into the cages of zeolite-Y by ion exchange, vapor impregnation, solid state diffusion, or direct synthesis within the cavities or channels of zeolites^21^. The luminescence of such zeolite-Y composite materials largely depends on the state of the luminescent centers (ions) in the cages of zeolite.

Among the family of inorganic luminescent materials, transition metal (TM) ions and lanthanide (Ln) ions are the common luminescent centers. Sensitization of luminescent centers is a popular phenomenon to enhance the luminescent efficiency or to realize specific wavelength excitation. Double dopants may have mutual influence on the spacial distribution of each other in the cage or cavity of zeolite-Y. Since the *d-d* transition of TM ions is susceptible to chemical environment due to no outer shell electrons and therefore no shield effect, while the *f-f* transition of Ln ions is less influenced due to the well shield effect by outer shell 5*s*5*p* electrons. Thus, TM and Ln ions codoped zeolite may serve as a good candidate for comparative study of their photoluminescence (PL), owing to the distinct photo-stimuli responses of TM and Ln ions to surrounding chemical enviorenments. Detailedly, Mn^2+^ ion is a well-studied luminescent center and commercially applicable in lighting and display^22^,^23^. The emission color of Mn^2+^ can be tuned from green to deep red^24^,^25^, depending on its coordinated chemical environment. It is recently reported by our group that two emission peaks at ~585 nm (visible) and ~770 nm (near infrared) in Mn^2+^ doped perovskite fluorides^26^, which are ascribed to the emission of isolated Mn^2+^ and the antiferromagnetic coupling within Mn^2+^-Mn^2+^ dimers, respectively. The latter could be controlled by concentration of Mn^2+^ dopant (i.e. the distance between Mn^2+^ ions) in a proper host lattice. Meanwhile, upconversion (UC) emission of Mn^2+^ could be realized by codoping Yb^3+^ in the Mn^2+^ doped materials^27^. It generally requires the vicinity of Mn^2+^-Yb^3+^ ions in the lattice for superexchange-interaction or cooperative sensitization based UC process, because there is a large gap between the first excited (emissive) state ^4^T_1_ and the ground state ^6^A_1_ for Mn^2+^ (normally >17,000 cm^−1^) while there is no metastable excited state above 10,000 cm^−1^ for Yb^3+^ ion^26^,^27^,^28^,^29^,^30^. Therefore, Mn^2+^-Yb^3+^ codopants may be an option in the zeolite-Y host to check their interaction and energy transfer involved.

This research utilizes various characterization techniques, including powder X-ray diffraction (XRD), Scanning Electronic Microscopy (SEM), BET measurement, Inductively Coupled Plasma Optical Emission Spectrometer (ICP-OES), PL spectroscopy, solid-state NMR, and Extended X-ray Absorption Fine Structure Spectroscopy (EXAFS), to check the Mn^2+^/Yb^3+^ interaction on the luminescence in Mn^2+^-Yb^3+^ exchanged zeolite-Y composites.

## Results and Discussion

### Phase and morphology

**Figure 1:**
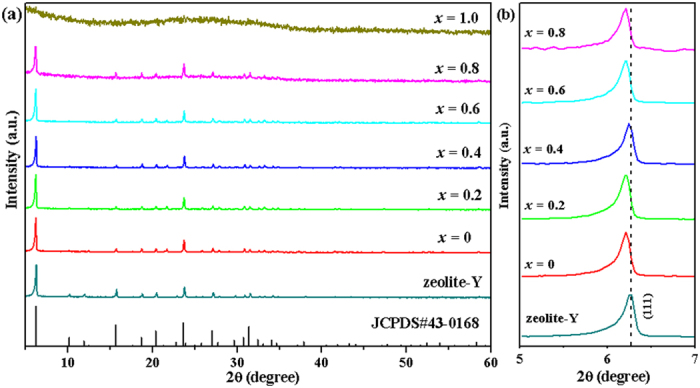
(**a**) XRD patterns of typical 0.2Yb, *x*Mn/zeolite-Y samples and the standard data for zeolite-Y (JCPDS#43-0168); (**b**) the enlarge (111) peaks for the samples.

Figure 1(a) displays the XRD patterns of 0.2Yb, *x*Mn/zeolite-Y (*x* = 0, 0.2, 0.4, 0.6, 0.8, 1.0; and it denotes the molar concentrations of Mn^2+^ ion in solution prior to exchange) samples. It can be seen that all the samples are in good agreement with the standard data of zeolite-Y (JCPDS#43-0168), except for the amorphous 0.2Yb,1.0Mn/zeolite-Y sample, suggesting the porous zeolite structure is maintained in all 0.2Yb, *x*Mn/zeolite-Y (*x* ≤ 0.8) samples., The gradually shift to the lower angles for the (111) diffraction peak of 0.2Yb, *x*Mn/zeolite-Y (*x* ≤ 0.8) at around 2θ = 6.2°, as seen in Fig. 1(b), indicates that Yb^3+^ and Mn^2+^ are successfully encapsulated in zeolite-Y and they enlarge the crystal lattice. Moreover, the diffraction peaks at around 10° and 12° becomes weak when Yb^3+^ and Mn^2+^ are incorporated and finally disappear with the increasing of Mn^2+^ contents. The result confirms that some ions entered the α cages of zeolite-Y^31^,^32^. With the increase of exchanged-ions contents, the crystallinity of 0.2Yb,*x*Mn/zeolite-Y samples is also calculated, which is shown in Fig. S1. When the concentration of Mn^2+^ in 0.2Yb, *x*Mn/zeolite-Y samples reaches 1.0 mol/L, the framework of zeolite-Y has collapsed and the sample turns to be glassy state. It can be inferred that the introduction of high contents of [MnCl] lowers the melting point of zeolites.

**Figure 2:**
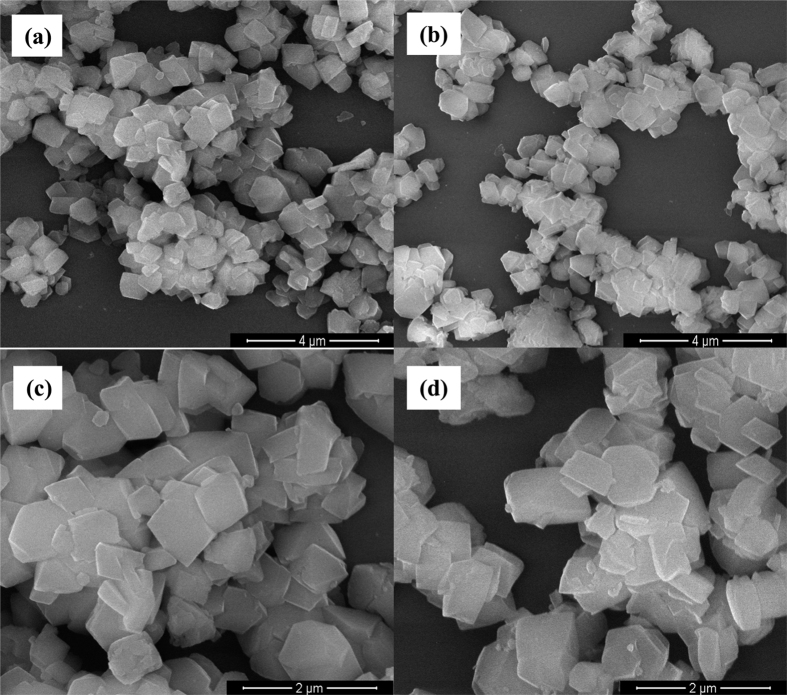
SEM images of some typical samples. (**a**,**c**) zeolite-Y calcined at 800 °C; (**b**,**d**) 0.2Yb, 0.8Mn/zeolite-Y calcined at 800 °C.

Since the immersion of zeolite-Y in the Yb^3+^ and Mn^2+^ solutions makes the Yb^3+^ and Mn^2+^ ions as well as the anion ions (such as Cl^−^) absorbed not only in the zeolite pores but also on the zeolite surface, which might result in unsmooth surface after the annealing treatment. Figure 2 displays the SEM images of some typical samples of zeolite-Y and 0.2Yb, 0.8Mn/zeolite-Y calcined at 800 °C. Apparently, the particles of the samples are angular with irregular shape and smooth surface, suggesting that the exchanged-ions may be mostly located inside the pores of zeolite-Y. Further evidences will be listed below.

### ICP and BET analysis

**Figure 3:**
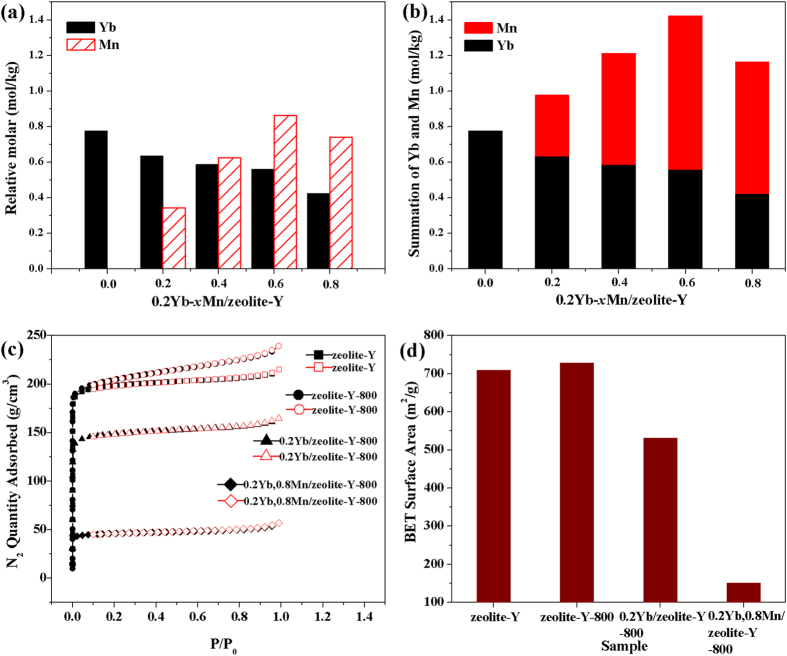
(**a**) Molar contents of Mn^2+^ and Yb^3+^ in 0.2Yb, *x*Mn/zeolite-Y; (**b**) summation of Yb^3+^ and Mn^2+^ molar contents in 0.2Yb, *x*Mn/zeolite-Y; (**c**) N_2_ adsorption-desorption isotherms; (**d**) specific surface area of the samples.

To evaluate the contents of exchanged Yb^3+^-Mn^2+^ ions in the 0.2Yb, *x*Mn/zeolite-Y(*x* ≤ 0.8) samples, ICP-OES measurement was conducted. Results in Fig. 3a show that with the increase of the designed concentration of Mn^2+^ solution, molar contents of Mn^2+^ gradually increases while that of Yb^3+^ declines slightly. Over all, the sum molar contents of Yb^3+^ and Mn^2+^ raise, as seen in Fig. 3b, suggesting that there are enough sites for both ions and there is a competition between them during the ion-exchanging reaction. Noticeably, the real molar ratio of Mn^2+^ to Yb^3+^ is smaller than that designed, which implies that Yb^3+^ preferably enter the cages of zeolite-Y.

Figure 3c shows the adsorption-desorption isotherms of some typical samples. The adsorption-desorption behaviour of each sample exhibits the typical porous characteristics. The maximum adsorption capacity of zeolite-Y without any treatment is 239.5 g·cm^−3^, while that of 0.2Yb, 0.8Mn/zeolite-Y is only 56.9 g·cm^−3^, indicating the filling behaviour of the zeolite-Y framework by the exchanged ions. The slight enhancement in the adsorption capacity of the zeolite-Y annealed at 800 °C compared to that of zeolite-Y without any treatment could be probably ascribed to the pre-desorption of water molecules for the former. The specific surface area of these samples exhibits analogous tendency in Fig. 3d. It can be inferred that the exchange ions have occupied the cation sites in cages of zeolite-Y, and high contents of exchanged ions may tend to block the pore structure of zeolites to prevent absorption of N_2_, causing the decreasing of specific surface area.

### Photoluminescence of *y*Yb, *x*Mn/zeolite-Y

**Figure 4:**
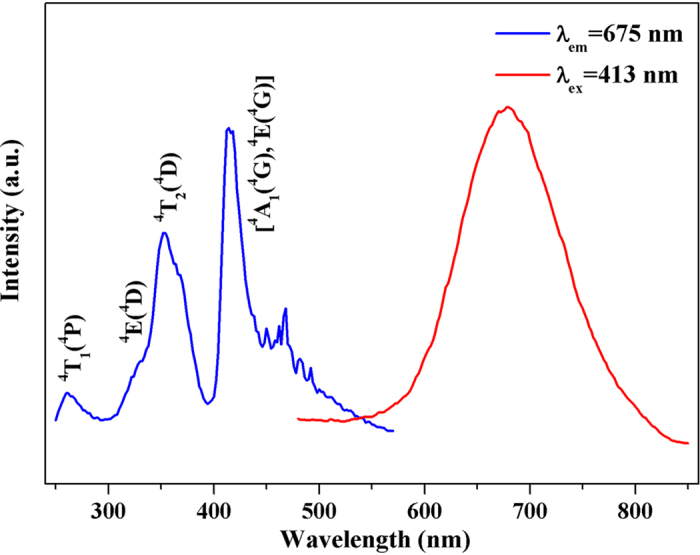
Excitation and emission spectra of 0.8Mn/zeolite-Y.

Figure 4 gives the excitation and emission spectra of 0.8 Mn/zeolite-Y. The red emission band located at 675 nm with a full width at half maximum (FWHM) of about 120 nm under the excitation of 413 nm is ascribed to the ^4^T_1_(^4^G) → ^6^A_1_(^6^S) transition of Mn^2+^ 33. While monitoring at 675 nm, a prominent excitation peak located at 413 nm can be attributed to the ^6^A_1_(^6^S) → ^4^A_1_(^4^G), ^4^E(^4^G) transition of Mn^2+^. And the band at around 350 nm, the shoulder peak at ~328 nm and the band at ~260 nm are assigned to the ^6^A_1_(^6^S) → ^4^T_2_(^4^D), ^6^A_1_(^6^S) → ^4^E(^4^D), and ^6^A_1_(^6^S) → ^4^T_1_(^4^P) transitions, respectively.

**Figure 5:**
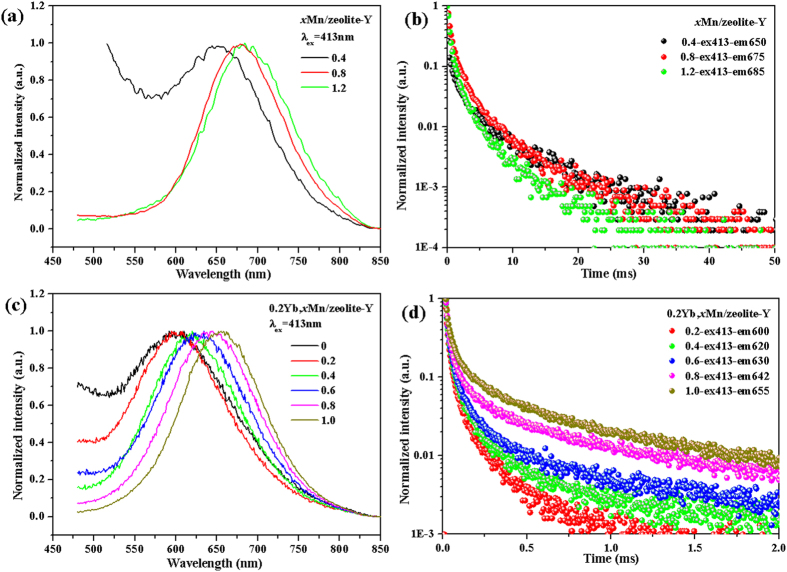
Emission spectra of *x*Mn/zeolite-Y (*x* = 0.4, 0.8, 1.2) (**a**) and 0.2Yb, *x*Mn/zeolite-Y (*x* = 0, 0.2, 0.4, 0.6, 0.8, 1.0) (**c**) upon excitation of 413 nm; (**b**,**d**) are their decay curves, respectively. Typically, 0.4-ex413-em650 in (**b**) denotes the excitation light of 413 nm with monitoring emission wavelength of 650 nm for the decay curves of 0.4 Mn/zeolite-Y.

The emission spectra of zeolite-Y doped with different Mn^2+^contents (*x* ≥ 0.4) excited by 413 nm are shown in Fig. 5a, and the luminescence of those samples with *x* < 0.4 is too weak to be detected. The emission peak shifts from 650 nm to 685 nm with increasing the Mn^2+^ contents in zeolite-Y, which is primarily owed to the interaction between Mn^2+^ ions with delocalized *d* electrons and multiple sites for Mn^2+^ ions in the cages of zeolite-Y^34^. In order to further investigate the luminescent process of Mn^2+^ in zeolite-Y, Fig. 5b depicted the decay curves. Figure 5c illustrates the emission spectra of 0.2Yb^3+^, *x*Mn^2+^/zeolite-Y (*x* = 0, 0.2, 0.4, 0.6, 0.8, 1.0) samples under the excitation of 413 nm. The emission band shows analogous shift from 595 nm to 655 nm for the samples with enhanced Mn^2+^ contents. Figure 5d shows the decay curves of 0.2Yb, *x*Mn/zeolite-Y (*x* = 0, 0.2, 0.4, 0.6, 0.8, 1.0) samples, in which the decay time of Mn^2+^ in these samples is prolonged with elevated Mn^2+^ contents. It is anomalous and quite interesting since the normal concentration quenching does not take place. Commonly, it would decay faster with the increase of Mn^2+^ content, owing to that there is energy transfer from Mn^2+^ (donor) to Yb^3+^ (acceptor) and concentration quenching among Mn^2+^ ions. Actually, the decay times for the samples in Fig. 5b are much longer than those in Fig. 5d, suggesting the quenching effects of Yb^3+^ ions as energy acceptor. Another cause may be that the occupancy preference of Yb^3+^ inside the cages of zeolite-Y (ICP-OES data) prevents the aggregation of Mn^2+^ ions in 0.2Yb, *x*Mn/zeolite-Y when the content of Mn^2+^ ions is low, resulting in more structural defects around Mn^2+^ ion. The structural defects would act as killing center of energy and make the Mn^2+^ luminescence decay faster(quenched). According to the most recent reports, cation-cation repulsion would prevent more cations to enter the pores of the zeolite^35^,^36^, especially for the higher valent Yb^3+^ and Mn^2+^ ions exchanging the original cation with +1 valence. With elevating the Mn^2+^ contents, aggregation in the atomic scale might take place^35^, which reduces the amount of defects. Further evidences will be listed in the following section. Herein, such aggregation of Mn^2+^ ions in atomic scale may not significantly cause the concentration quenching. Also it should be noticed that the real content of Mn^2+^ for the same nominal Mn^2+^ contents in Fig. 5b,d should be not actually the same due to the preferred occupancy of Yb^3+^ ions and the cation-cation repulsion in zeolite. This is also the cause of different emission wavelengths for those samples with same *x*.

**Figure 6:**
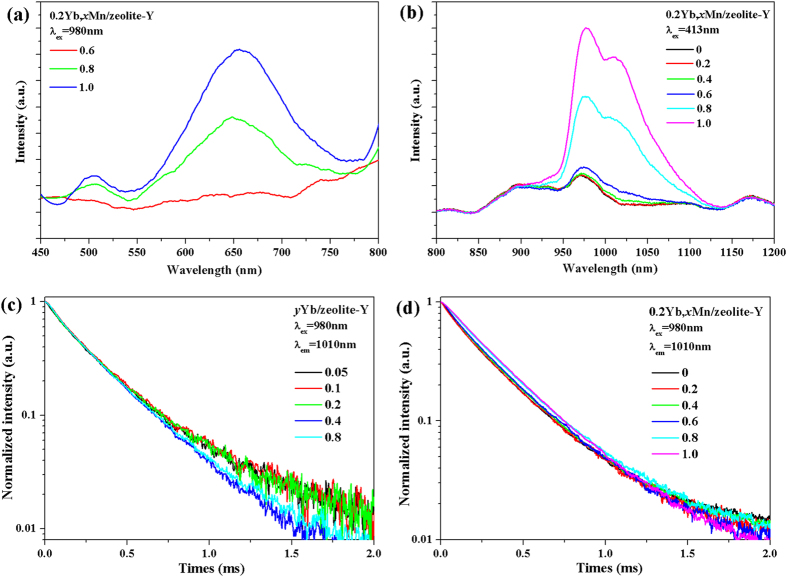
(**a**) UC luminescence of 0.2Yb, *x*Mn/zeolite-Y (*x* = 0.6, 0.8, 1.0) under excitation of 980 nm laser diode; (**b**) near infrared luminescence of Yb^3+^ in 0.2Yb, *x*Mn/zeolite-Y (*x* = 0.2, 0.4, 0.6, 0.8, 1.0) samples under excitation of 413 nm xenon light; decay curves of Yb^3+^ emission in (**c**) *y*Yb/ zeolite-Y (*y* = 0.05, 0.1, 0.2, 0.4, 0.8) samples and (**d**) 0.2Yb, *x*Mn/ zeolite-Y (*x* = 0, 0.2, 0.4, 0.6, 0.8, 1.0) samples upon excitation of 980 nm laser diode.

0.2Yb, *x*Mn/zeolite-Y (*x* = 0.8, 1.0) samples could show UC luminescence under excitation of 980 nm laser diode, as seen in Fig. 6a, while those samples (*x* = 0, 0.2, 0.4, 0.6) exhibit no apparent UC emissions. Since there is a large gap between the first excited state of ^4^T_1_ and ground state of ^6^A_1_ for Mn^2+^ (normally > 17,000 cm^−1^) and there is no metastable excited state above 10,000 cm^−1^ for Yb^3+^ ion^26^,^27^,^28^,^29^,^30^, it normally requires the vicinity of Mn^2+^-Yb^3+^ ions in the lattice for super exchange-interaction or cooperative sensitization based UC process. Therefore, when Mn^2+^ content is high enough (*x* ≥ 0.8), the Mn^2+^ ions tend to show up at the neighbor of Yb^3+^ ions. Furthermore, an UC emission band located at 505 nm, which is attributed to the UC process of Yb^3+^-Yb^3+^ ion pair, appears when *x* ≥ 0.8. It also manifests that introduction of high Mn^2+^ content would make Yb^3+^ ions aggregate. Interestingly, the near infrared luminescence ^2^F_5/2_ → ^2^F_7/2_ of Yb^3+^ in 0.2Yb, *x*Mn/zeolite-Y (*x* = 0.8, 1.0) samples under excitation of Mn^2+^ are apparently observed in Fig. 6b, suggesting that there is energy transfer between Mn^2+^ and Yb^3+^ despite of the large gap between ^4^T_1_ of Mn^2+^ and ^2^F_5/2_ of Yb^3+^. It also implies the distance between Mn^2+^and Yb^3+^ is shortened enough for the energy transfer took place for samples with *x* ≥ 0.8. The decay curves of Yb^3+^ in *y*Yb/zeolite-Y (*y* = 0.05, 0.1, 0.2, 0.4, 0.8) and 0.2Yb, *x*Mn/zeolite-Y (*x* = 0.2, 0.4, 0.6, 0.8, 1.0) samples in Fig. 6c,d show little change, which is quite different from that of Mn^2+^. But by focusing in detail, the decay curves show more linear when increasing the Yb^3+^ contents or the Mn^2+^ contents, again manifesting the aggregation of Yb^3+^ ions. It is also consistent with the luminescence behavior in Fig. 6a,b. Accordingly, it can be inferred that the aggregation of Yb^3+^ ions reduces defects, which makes the decay curves of Yb^3+^ ion with few defects surrounded exhibit single-exponential behaviour.

### Solid state NMR and EXAFS analysis

**Figure 7:**
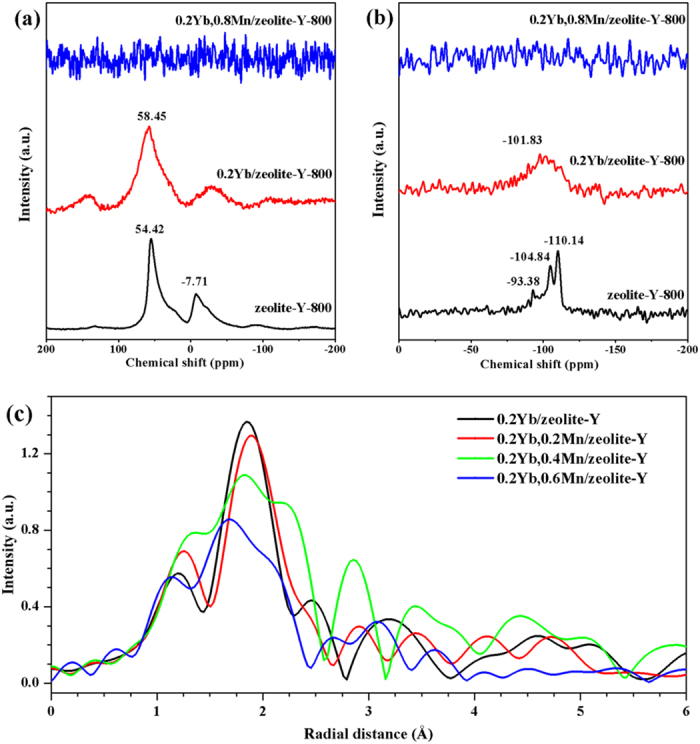
(**a**) ^27^Al NMR spectra, (**b**) ^29^Si NMR spectra, (**c**) Fourier transformed EXAFS spectra of Yb L-edge of 0.2Yb, *x*Mn/zeolite-Y samples.

In order to detect the structure variation of Yb^3+^, Mn^2+^ codoped zeolite-Y, the ^27^Al and ^29^Si NMR spectroscopy of some typical samples were measured and shown in Fig. 7a,b. As for the ^27^Al NMR spectra, the peak located at around 54 ppm is attributed to the framework tetrahedral Al in zeolite-Y, while the peak located at around −7.7 ppm is attributed to the non-framework hexahedral Al^37^. It can be seen from Fig. 7a that the both peaks are weaker and broadened when doped with Yb^3+^ ions. And the latter shows an apparent shift to the low chemical shift direction, suggesting that there is an electron shielding effect of Yb^3+^ on the non-framework ^27^Al nuclear. While, no resonant peak can be observed in 0.2Yb,0.8Mn/zeolite-Y sample, implying that the *d* electrons of Mn^2+^ may cause a large spin relaxation of ^27^Al nuclear. For the ^29^Si spectra, obvious peaks at -110.14 ppm, -104.84 ppm and -93.38 ppm are observed in Fig. 7b, which are ascribed to the Si0 (no Al ion connected), Si1 (connected with 1Al ion), Si3 (connected with 3Al ions)^38^,^39^, respectively. These peaks are merged together and appear to be one broad peak for the 0.2Yb/zeolite-Y, and the peak at around −110 ppm become much weaker, which is also ascribed to the strong shielding effect of Yb^3+^ on Si0 (no Al ion connected, probably the Si at the inner surface of pores). It also shows no signals for 0.2Yb, 0.8Mn/zeolite-Y, owing to the strong spin relaxation effect on Si caused by electron of Mn^2+^. The results of Yb^3+^ singly doped zeolite-Y compared to that of pure zeolite-Y suggest that Yb^3+^ would dispersively occupy the cation sites in the zeolite-Y pores.

Figure 7c shows the EXAFS spectra of Yb L-edge of 0.2Yb, *x*Mn/ zeolite-Y (*x* = 0, 0.2, 0.4, 0.6) samples. Owing to lack of specific structure model for the complex composite, we are unable to fit the data with Fourier-transform. Therefore, the EXAFS data is qualitatively analyzed. The peak located at around 2 Å can be attributed to the coordination peak of Yb-O, then the peak located at around 2.2 Å could be explained by the coordination of Yb^3+^-Cl^− ^40 (Cl^−^ is introduced by MnCl_2_·4H_2_O, and the effective ionic radius of Cl^−^ is 1.81 Å, larger than 1.35 Å of O^2− ^41). It can be seen that the coordination peak of Yb-O becomes weaker while that of Yb-Cl tends to show up and gradually be enhanced as the content of Mn^2+^ increases. The Cl^−^ introduced by [MnCl] presents at the vicinity of Yb^3+^ only beyond certain contents of Mn^2+^ in the samples, which is ascribed to that the cation-cation repulsion hinders a new cation to be absorbed inside the cavity of the zeolite^35^,^36^. However, both the two peaks shift to the smaller value when the Mn^2+^ content *x* reaches 0.6, which is likely caused by the partial collapse or densification of zeolite-Y framework. It is also consistent with the discussion above.

### Luminescence mechanisms

**Figure 8:**
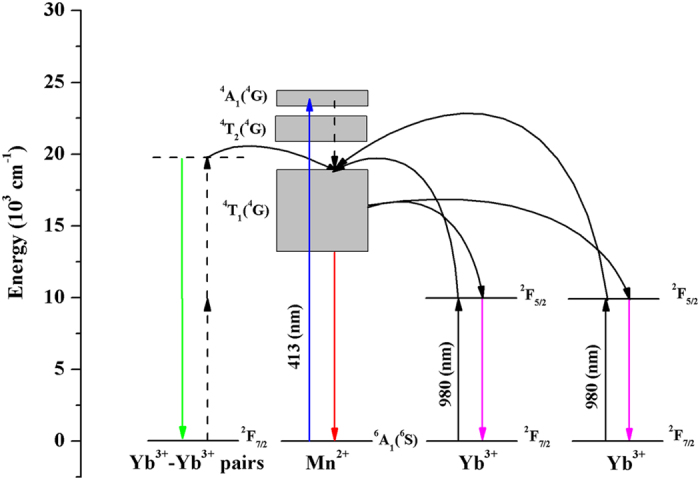
UC and Stokes luminescence mechanisms of Yb, Mn/zeolite-Y.

The UC and stokes luminescence mechanisms of Yb^3+^-Mn^2+^ are proposed in zeolite-Y and schematically illustrated in Fig. 8. For the Stokes luminescence of Mn^2+^, the 413 nm light excites an electron of Mn^2+^ from ground state ^6^A_1_(^6^S) to the ^4^A_1_(^4^G) level, sequentially relax nonradiatively to the ^4^T_1_(^4^G) level and then to the ^6^A_1_(^6^S) level with the emission ranged from 595 nm to 655 nm. For Stokes luminescence of Yb^3+^, it can either be excited by 980 nm laser or by energy transfer from the nearby Mn^2+^ ion via ^2^F_5/2_ level. For UC luminescence of Yb^3+^, it is originated from the coupled Yb^3+^-Yb^3+^ pairs by simultaneously absorbed two 980 nm photons. While for the UC emission of Mn^2+^, a cooperative sensitization process from two excited Yb^3+^ ions pumped by 980 nm laser to the ^4^T_1_(^4^G) level of Mn^2+^, leading to a red emission of ~650 nm when further depopulated to the ^6^A_1_(^6^S) level.

In summary, Mn^2+^-Yb^3+^ codoped zeolite-Y are synthesized by the simple ion-exchanging method, and the interaction between the two exchanged ions is discussed. Results show that Yb^3+^ is prior to occupy the cation sites in zeolite channels. Incorporation of Mn^2+^ and Yb^3+^ ions in zeolites simultaneously would promote the mutual aggregation of each ion according to the luminescence data, because it generally requires the vicinity of Mn^2+^-Yb^3+^ ions in the zeolite-Y for the energy transfer based luminescence. The EXAFS data also support this point. The research offers a perspective on the emissive zeolite with two distinct and interacted luminescent centers.

## Methods

### Synthesis

Powder samples of Mn^2+^ doped zeolite-Y (denoted as *x*Mn/ zeolite-Y, *x* = 0.4, 0.8, 1.2 mol/L), Yb^3+^ doped zeolite-Y (denoted as *y*Yb/zeolite-Y, *y* = 0.05, 0.1, 0.2, 0.4, 0.8 mol/L), and those codoped with both Mn^2+^ and Yb^3+^ (denoted as *y*Yb^3+^, *x*Mn^2+^/zeolite-Y, *y* = 0.2 mol/L, *x* = 0, 0.2, 0.4, 0.6, 0.8, 1.0 mol/L) were prepared by ion-exchange reaction method. The raw materials zeolite-Y (SiO_2_/Al_2_O_3_ = 5.1), Yb(NO_3_)_3_·5H_2_O (99.9%) and MnCl_2_·4H_2_O (99.99%) were purchased from Alfa Aesar. The zeolites were initially stirred in the aqueous solution of MnCl_2_·4H_2_O and Yb(NO_3_)_3_·5H_2_O at 80 °C for 36 h. Then the resulting products were centrifugated and washed with deionized water. After several times of washing and centrifugation, the products were kept in the oven at 80 °C for 12 h for drying. Finally, they were placed into an alumina crucible and sintered at 800 °C for 1 h under a reductive atmosphere (5%H_2_ + 95%N_2_) in a furnace.

### Characterizations

The phase of the samples was characterized by a Rigaku D/max-IIIA X-ray diffractometer with Cu-Kα radiation (λ = 1.5418 Å). The morphology of samples was detected by field emission SEM (FEI, Nova Nano SEM 430). The contents of Mn^2+^ and Yb^3+^ in zeolites were determined by ICP-OES (Varian, 720-ES). The specific surface area of samples was checked by BET measurement (Quantachrome, NOVA2000e). Solid-state ^27^Al and ^29^Si NMR of the samples were measured on Bruker BioSpin, Bruker AVANCE IIIT 600HD. The emission, excitation spectra and decay curves were recorded using a fluorescence spectrophotometer (Edinburgh Instruments, FLS920) with Xe lamp as the light source. The emission spectra excited by a 980 nm laser diode (Shenzhen Leo-photoelectric Co., LTD) were recorded on an iHR320 fluorescence spectro-fluorometer (Horiba Jobin-Yvon Co.) equipped with an R928 photomultiplier tube (PMT). EXAFS data (Yb L-edge) of the samples were collected at 16BM-D, Advanced Phonon Source, Argonne National Laboratory. The data were processed and fitted with the program Athena^42^.

## Additional Information

**How to cite this article**: Ye, S. *et al*. Interaction between the exchanged Mn^2+^ and Yb^3+^ ions confined in zeolite-Y and their luminescence behaviours. *Sci. Rep.* **7**, 46219; doi: 10.1038/srep46219 (2017).

**Publisher's note:** Springer Nature remains neutral with regard to jurisdictional claims in published maps and institutional affiliations.

## Supplementary Material

Supplementary Information
