# Supplementary material for: Interaction between the exchanged Mn2+ and Yb3+ ions confined in zeolite-Y and their luminescence behaviours
Source: Sci Rep. 2017 Apr 10;7:46219. doi: 10.1038/srep46219 (PMC5385532; doi:10.1038/srep46219)
Supplement: Supplementary Information [file srep46219-s1.doc]

**Supporting information**

**Interaction between the exchanged Mn^2+^ and Yb^3+^ ions confined in zeolite-Y and their luminescence behaviours**

**Shi Ye*****[[1]](#footnote-2), Jiayi Sun^1^, Xiong Yi^1^, Yonggang Wang^2^ & Qinyuan ZhangError: Reference source not found**


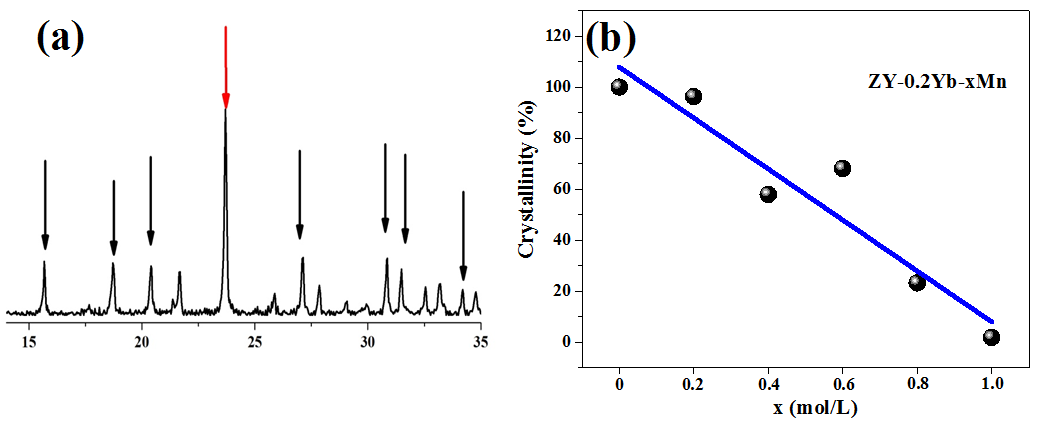


**Fig.S1** (a)The peaks calculated for the crystallinity in the XRD pattern; (b) Crystallinity of ZY:0.2Yb^3+^-xMn^2+^ (x=0.0,0.2,0.4,0.6,0.8,1.0) samples

According to the standard of petrochemical industry in PRC, the crystallinity of Yb^3+^-Mn^2+^ codoped zeolites was calculated and shown in Fig.S1. We calculated the sum of the integral area of the eight peaks which are marked in Fig.S1a. And the full width at half maximum (FWHM) of (533) diffraction peak of the samples was also measured and the crystallinity was calculated by the following equation:

X_R_ stands for the crystallinity of ZY:0.2Yb^3+^( which is without Mn^2+^ doped, and the crystallinity is supposed to be 100%); W_R_ stands for the FWHM of (533) diffraction peak. It can be observed that the crystallinity of samples showed a decreasing trend as Mn^2+^ concentration increasing, which is agreement with our discussion about the structure change of zeolite-Y.

1. State Key Lab of Luminescent Materials and Devices, and Guangdong Provincial Key Laboratory of Fiber Laser Materials and Applied Techniques, South China University of Technology, Guangzhou 510641, China. ^2^High Pressure Synergetic Consortium (HPSynC), Geophysical Laboratory, Carnegie Institution of Washington, Argonne, Illinois 60439, United States. *Correspondence and requests for materials should be addressed to S. Y. (email: msyes@scut.edu.cn) [↑](#footnote-ref-2)
